# Emergence and Genomic Characterization of Neisseria gonorrhoeae Isolates with High Levels of Ceftriaxone and Azithromycin Resistance in Guangdong, China, from 2016 to 2019

**DOI:** 10.1128/spectrum.01570-22

**Published:** 2022-11-15

**Authors:** Xiaomian Lin, Wentao Chen, Yuqi Yu, Yinyuan Lan, Qinghui Xie, Yiwen Liao, Xingzhong Wu, Sanmei Tang, Xiaolin Qin, Heping Zheng

**Affiliations:** a Dermatology Hospital, Southern Medical University, Guangzhou, Guangdong, China; b Guangzhou Key Laboratory for Sexually Transmitted Disease Control, Guangzhou, Guangdong, China; ATCC

**Keywords:** *N. gonorrhoeae*, dual resistance, *penA*-60.001, 23S rRNA, genome analysis, A2735, F89, FC428

## Abstract

Currently, antibiotic resistance (especially ceftriaxone and azithromycin dual resistance) in Neisseria gonorrhoeae is the main obstacle affecting the efficacy of treatment. As analysis of drug sensitivity, molecular features, and dissemination of dual-resistant strains is important for gonococcal prevention and control, MIC, genotyping, and genome analysis were conducted to reveal the molecular characteristics and phylogeny of N. gonorrhoeae isolates. During 2016 to 2019, 5 out of 4,113 strains were defined as dual-resistant clones, with ceftriaxone MICs of 0.25 to ≥1 mg/L and azithromycin MICs of 2 to ≥2,048 mg/L. In particular, two strains with a ceftriaxone MIC above 0.5 mg/L were characterized as *penA*-60.001 FC428-related clones, and two isolates with a high-level azithromycin MIC above 1,024 mg/L featuring a 23S rRNA mutation were identified. Furthermore, phylogenetic analysis confirmed that the dual-resistant strains were closer to the evolutionary origin of F89 in France, global FC428-related clones, and high-level dual-resistant clones in Australia and the United Kingdom. Dual-resistant strains, including FC428-related clones and high-level azithromycin-resistant clones, have circulated in Guangdong, China. The ability of laboratories to perform real-time drug susceptibility and genetic analyses should be strengthened to monitor the spread of threatening strains.

**IMPORTANCE** Here, we report five sporadic dual-resistant isolates, including FC428-related ceftriaxone-resistant clones with MICs of ≥0.5 mg/L and high-level azithromycin resistance with MICs of ≥1,024 mg/L. This study highlights that dual-resistant clones with the same evolutionary origin as FC428, A2735, and F89 have circulated in Guangdong, China, which suggests that the capacity for antibiotic resistance testing and genome analysis should be strengthened in daily epidemiological surveillance.

## INTRODUCTION

Neisseria gonorrhoeae is the second most common sexually transmitted infection (STI) and is predicted to have an annual global incidence of 82.4 million new cases (1). Among these, 40 to 50% of the estimated cases will occur in the Western Pacific Region; China accounts for the most cases and contributes substantially to the number of people with gonorrhea (2). In the absence of vaccines and new drugs, the treatment of gonorrhea relies only on antibiotics such as ceftriaxone, which is the last remaining effective option for first-line antimicrobial monotherapy. With the widespread dissemination of ceftriaxone-resistant clones, ceftriaxone efficacy has been hampered (3). To limit the spread of antimicrobial resistance (AMR), dual therapy (azithromycin plus ceftriaxone) for gonorrhea was recommended by the Centers for Disease Control and Prevention (CDC) in 2010 (4), the European Committee in 2012 (5), and the World Health Organization (WHO) in 2016 (6).

However, since the introduction of dual therapy, clones resistant to both ceftriaxone and azithromycin have been isolated sporadically. In 2016, a patient from the United Kingdom first failed to dual treatment, calling the efficacy of dual therapy into question (7). Concerningly, the dual-resistant strain F90 was subsequently discovered in France in 2017 (8); high-level ceftriaxone (MIC ≥ 0.25 mg/L) and azithromycin (MIC ≥ 256 mg/L) resistance clones found in England in 2018 (G97687 and G7944) (9) and in Australia (A2543 and A2735) (10) have been a major public health concern. In Changsha, an N. gonorrhoeae strain named GC250 with resistance to both ceftriaxone (0.5 mg/L) and azithromycin (2 mg/L) was first isolated in China in 2018 (11). Then, YL201, with high-level ceftriaxone resistance (0.75 mg/L) and moderate-level azithromycin resistance (12 mg/L), was identified in Shenzhen in 2020 (12). Here, we report another five dual-resistant N. gonorrhoeae strains in Guangdong Province, some of which show the first incidence of high-level resistance to both ceftriaxone (MIC ≥ 0.25 mg/L) and azithromycin (MIC ≥ 1,024 mg/L) in China. As widespread circulation of dual-resistant strains might increase the burden of gonorrhea treatment, molecular surveillance needs to be strengthened before the application of new drugs.

## RESULTS

### Antibiotic resistance surveillance and the occurrence of dual-resistant N. gonorrhoeae strains.

Based on antimicrobial susceptibility testing (AST) of 4,113 N. gonorrhoeae strains, we identified five dual-resistant strains. As shown in Table 1, the five strains exhibited resistance to azithromycin (MIC = 2 to ≥2,048 mg/L), cefixime (MIC = 0.25 to ≥1 mg/L), ceftriaxone (MIC = 0.25 to ≥1 mg/L), penicillin (MIC = 1 to ≥32 mg/L), tetracycline (MIC = 2 to ≥32 mg/L), and ciprofloxacin (MIC ≥ 16 mg/L) but susceptibility to spectinomycin (MIC = 8 to 32 mg/L). With these strains, we reveal the first two (SS43 and SS76) high-level azithromycin-resistant isolates (MIC ≥ 1,024 mg/L) that also exhibit resistance to ceftriaxone with an MIC of 0.25 mg/L. Furthermore, two clones (DG17067 and GD69), identified as having high-level ceftriaxone (MIC ≥ 0.5 mg/L) and low-level azithromycin (MIC‗2 mg/L) resistance, were identified in Guangdong, China. The dissemination of dual-resistant strains can seriously affect the efficacy of dual therapy (ceftriaxone plus azithromycin), and there is a need for improved surveillance of gonococcal AMR.

**TABLE 1:** MICs of antibiotics against dual-resistant isolates^*a*^

Strain	Yr	City	MIC (mg/L)						
			AZM	CFM	CRO	PEN	TET	CIP	SPT
SS36	2016	Guangzhou	2	0.25	0.25	1	4	≥16	32
SS43	2016	Guangzhou	≥2,048	0.5	0.25	8	4	≥16	16
SS76	2019	Guangzhou	1,024	0.25	0.25	8	≥32	≥16	16
DG17067	2017	Dongguan	2	≥1	≥1	1	2	≥16	8
GD69	2018	Guangzhou	2	≥1	0.5	≥32	4	≥16	16

a AZM, azithromycin; CFM, cefixime; CRO, ceftriaxone; PEN, penicillin; TET, tetracycline; CIP, ciprofloxacin; SPT, spectinomycin.

### Molecular characterization of AMR in dual-resistant N. gonorrhoeae strains.

A complete understanding of the potential resistance mechanisms associated with the development of dual-resistant gonorrhea requires the ability to adequately molecularly characterize strains. In addition to non-sequence-based typing methods such as AST, sequence-based methods such as genotyping and genome analysis are also utilized to compare isolates of interest to reveal molecular features (13). As shown in Table 2, using N. gonorrhoeae multiantigen sequence typing (ngMAST), we divided the five clones into four sequence types (STs), including two ngMAST-ST1866 clones and three novel subtypes, and differences were mainly identified in the single-nucleotide polymorphisms (SNPs) in the *porB* and *tbpB* genes. For multilocus sequence typing (MLST), two out of five dual-resistant clones featured MLST-ST1901, and the others were characterized as MLST-ST7365, MLST-ST1903, and MLST-ST10899 (Table 3). Additionally, N. gonorrhoeae sequence typing for antimicrobial resistance (ngSTAR) resulted in only two strains with ngSTAR classification (ngSTAR-ST501 and ngSTAR-ST2502), while the others have not been identified to date (Table 4).

**TABLE 2:** ngMAST of dual-resistant isolates^*a*^

Strain	Sequence type of gene:		ngMAST
	*porB*	*tbpB*	
SS36	700	1308	NA
SS43	581	33	1866
SS76	581	33	1866
DG17067	NA	21	NA
GD69	9800	21	NA

a NA, unknown sequence type.

**TABLE 3:** MLST of dual-resistant isolates^*a*^

Strain	Sequence type of gene:							MLST
	*abcZ*	*aroE*	*fumC*	*adk*	*gdh*	*pdhC*	*pgm*	
SS36	109	170	111	39	148	153	65	1901
SS43	126	170	158	39	148	153	65	10899
SS76	109	170	111	39	148	153	65	1901
DG17067	126	67	111	39	148	153	65	7365
GD69	126	67	157	39	148	153	65	1903

a NA, unknown sequence type.

**TABLE 4:** ngSTAR of dual-resistant isolates^*a*^

Strain	Sequence type of gene:							ngSTAR
	*penA*	*mtrR*	*porB*	*ponA*	*gyrA*	*parC*	23S rRNA	
SS36	18.001	36	8	1	2	24	100	501
SS43	2.002	319	8	1	7	3	1	2502
SS76	NA	1	8	1	7	3	NA	NA
DG17067	60.001	1	12	1	7	3	100	NA
GD69	60.001	1	12	1	21	3	100	NA

a NA, unknown sequence type.

As ngSTAR is based on the nucleotide sequences of seven genes (*penA*, *mtrR*, *porB*, *ponA*, *gyrA*, *parC*, and 23S rRNA) associated with AMR, we further analyzed the gene mutation profiles in Table 5. As shown, strains DG17067 and GD69, with a ceftriaxone MIC of ≧0.5 mg/L, carried the type LX mosaic *penA*-60.001 allele, featuring gene mutations such as D345A, F504L, N512Y, and G545S. Unexpectedly, strains (SS36 and SS43) featuring the nonmosaic *penA*-18.001 and *penA-*2.002 alleles also exhibited resistance to ceftriaxone, and a new type of *penA* allele existed in isolate SS36. All isolates had the −35A deletion in the *mtrR* promoter region and mutations in *porB* (G120K, A121D, or A121G), *ponA* (L421P), *gyrA* (S91F, D95N, or D95A), and *parC* (S87I or S87R), which were all associated with the overexpression of the MtrCDE efflux pump, decreased influx of the porin channel, plasmid-mediated resistance, and a reduced rate of penicillin acylation. In addition, isolate SS43, with an azithromycin MIC of over 1,024 mg/L, was characterized as having 23S rRNA-1 mutant alleles, which encoded A2059G alterations and were related to high-level resistance and *in vivo* biological fitness (14). Except for SS76, which harbored a novel allele with high-level azithromycin resistance, the others had wild-type 23S rRNA sequences. According to the ngSTAR mutant gene profiles, all isolates exhibited resistance to azithromycin, cephalosporin, penicillin, ciprofloxacin, and tetracycline, which is consistent with previous reports (15–17).

**TABLE 5:** Antibiotic resistance gene profiles identified among dual-resistant strains

Isolate	AMR profile in gene:							AMR plasmid
	NEIS1753 (*penA*)	*mtrR*	NG_porB	NG_ponA	NG_gyrA	NG_parC	NG_23S	
SS36	Mutations: A501T, F504L; type XVIII nonmosaic *penA* allele: 18.001	−35ADel; mutation: A39T	Mutations: G120K, A121D	Mutation: L421P	Mutations: S91F, D95N	Mutation: S87I	Wild_type	*bla*_TEM-1_
SS43	Mutations: F504L; type II nonmosaic *penA* allele: 2.002	−35ADel; mutation: G45D	Mutations: G120K, A121D	Mutation: L421P	Mutations: S91F, D95A	Mutation: S87R	A2059G	*bla*_TEM-1_, *tet*(M)
SS76	New allele	−35ADel	Mutations: G120K, A121D	Mutation: L421P	Mutations: S91F, D95A	Mutation: S87R	New allele	*bla*_TEM-1_
DG17067	Mutations: D345A, F504L, N512Y, G545S; type LX mosaic *penA* allele: 60.001	−35ADel	Mutations: G120K, A121G	Mutation: L421P	Mutations: S91F, D95A	Mutation: S87R	Wild_type	No detection
GD69	Mutations: D345A, F504L, N512Y, G545S; type LX mosaic *penA* allele: 60.001	−35ADel	Mutations: G120K, A121G	Mutation: L421P	Mutations: S91F, D95A	Mutation: S87R	Wild_type	No detection

Further, to clarify the underlying AMR molecules, we used ABRicate for analysis. As shown in Table 6, the five dual-resistant strains harbored a set of virulence factors, including ABC transporters (*farA* and *farB*), the antigen 85 proteins (*fbpA*, *fbpB*, and *fbpC*), a hemoglobin receptor gene (*hmbR*), outer membrane proteins (*hpuA* and *hpuB*), dLdH catalase (*katA*), *kdtA*, a lactoferrin import receptor (*lbpA*), gonococcal lipooligosaccharide-related genes (*lgtA*, *lgtF*, and *lgtG*), the MntABC system, Pil genes (*msrAB*/*pilB*, *pilD*, *pilF-K*, *pilM-P*, *pilS-X*, and *pilZ*), an *mtrC*-*mtrD*-*mtrE* efflux pump, surface protein A (*nspA*), the porin gene *porA*, a DNA repair gene (*recN*), lipopolysaccharide biosynthesis genes (*rfaC*, *rfaF*, and *rfaK*), and a transferrin receptor (*tbpA*), which might have a huge impact on the infection and dissemination process of N. gonorrhoeae strains. In addition, not all dual-resistant clones carried the genes *lbpB*, *lgtB*, *lgtE*, *opa*, or *pilC*, so the role of these virulence factors needs to be further evaluated.

**TABLE 6:** Virulence factor analysis of dual-resistant strains

Virulence factor	Coverage (%) for genes^*a*^				
	SS36	SS43	SS76	DG17067	GD69
*farA*	100	100	100	100	100
*farB*	99.8	99.8	99.8	99.8	99.8
*fbpA*	100	100	100	100	100
*fbpB*	100	100	100	100	100
*fbpC*	100	100	100	100	100
*hmbR*	99.83	99.83	99.83	99.83	99.83
*hpuA*	98.23	98.14	98.23	98.32	98.32
*hpuB*	99.88	99.88	99.88	99.88	99.88
*katA*	100	100	100	100	100
*kdtA*/*waaA*	100	100	100	100	100
*lbpA*	100	100	100	81.43	81.43
*lbpB*	97.83	97.43	97.79	-	-
*lgtA*	99.52	99.24	99.24	99.52	99.43
*lgtB*	100	100	100	-	-
*lgtE*	100	100	100	100	-
*lgtF*	100	100	100	100	100
*lgtG*	95.35	95.45	95.35	95.45	95.26
*mntA*	100	100	100	100	100
*mntB*	100	100	100	100	100
*mntC*	100	100	100	100	100
*msrAB* (*pilB*)	100	100	100	100	100
*mtrC*	100	100	100	100	100
*mtrD*	100	100	100	100	100
*mtrE*	100	100	100	100	100
*nspA*	100	100	100	100	100
*opa*	94.73	92.03	-	91.42	-
*pilC*	-	-	82.47	98.93	-
*pilD*	99.3	99.3	99.3	99.3	99.3
*pilF*	100	100	100	100	100
*pilG*	100	100	100	100	100
*pilH*	93.57	93.57	93.57	93.57	93.57
*pilI*	100	100	99.51	100	100
*pilJ*	99.68	99.04	99.04	99.68	99.68
*pilK*	100	100	100	100	100
*pilM*	100	100	100	100	100
*pilN*	100	100	100	100	100
*pilO*	100	100	100	100	100
*pilP*	100	100	100	100	100
*pilS*	91.34	99.29	93.07	99.35	99.35
*pilT*	100	100	100	100	100
*pilT2*	100	100	100	100	100
*pilU*	100	100	100	100	100
*pilV*	99.49	99.49	99.49	100	100
*pilW*	100	100	100	100	100
*pilX*	99.79	99.79	99.79	99.79	99.79
*pilZ*	100	100	100	100	100
*porA*	97.12	97.03	97.12	97.03	97.03
*recN*	100	100	100	100	100
*rfaC*	100	100	100	100	100
*rfaF*	100	100	100	100	100
*rfaK*	100	100	100	100	100
*tbpA*	99.67	99.02	99.56	99.67	99.67

a “-” indicate the absence of a gene.

In conclusion, genome characterization of N. gonorrhoeae isolates can accurately reflect the antibiotic phenotype in real time, which can make up for the lag of AST and help with monitoring the prevalence and dissemination of dangerous strains.

### Phylogenomic analysis of dual-resistant N. gonorrhoeae strains.

**FIG 1:**
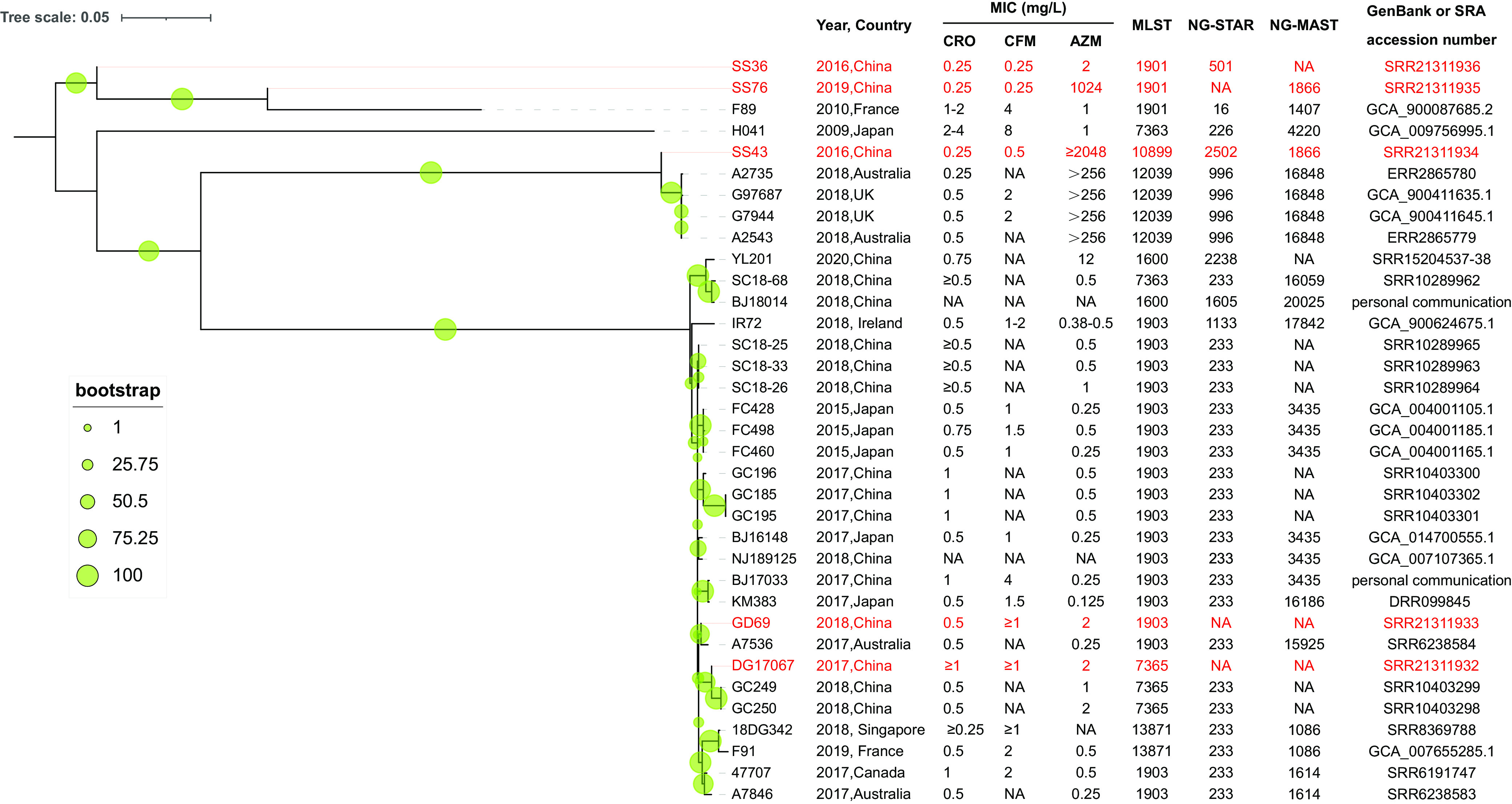
Maximum likelihood (ML) phylogeny of the dual-resistant strains. The ML phylogeny was constructed based on 1,558 single-nucleotide variants (SNVs) in nonrecombination regions along the whole genome. The bootstrap values are labeled as green gradient dots on the branches. The tree scale bar indicates the average number of single-nucleotide polymorphisms (SNPs) per site.

Because revealing the evolutionary origins of dual-resistant strains is essential for N. gonorrhoeae control, phylogenomic analysis was performed. As shown in Fig. 1, the five dual-resistant strains were divided into three clusters and had different evolutionary trajectories. In the FC428-related cluster, two *penA*-60.001 clones, DG17067 and GD69, were highly similar to those found in Changsha, China (2018), and Australia (2017). For the two extremely azithromycin-resistant isolates, SS43 (found in 2016) was correlated with high-level dual resistance strains (2018; Australia and UK), and SS76 had the same evolutionary origin as F89, isolated in France in 2010. SS36, which was not homologous to any existing dual-resistant isolates, alone constituted an evolutionary branch. Ceftriaxone-resistant clones (especially FC428-, F89-, and A2735-related clones) have circulated in Guangdong, China.

## DISCUSSION

Gonorrhea remains a major public health concern globally, and the numbers of dual-resistant N. gonorrhoeae strains have been increasing (18). Our study is the first report of high-level dual-resistant strains in Guangdong, China. As shown, two FC428-related *penA*-60.001 clones (GD69 and DG17067) that had acquired moderate-level resistance to azithromycin were found locally; the strains are similar to YL201, which was reported in Shenzhen, Guangdong (12). Although the three isolates were all FC428-related clones, phylogenetic analysis showed that the origins of these strains were significantly different (Fig. 1). Specifically, GD69 is in the same evolutionary node as the Australian strain A7536, DG17067 is more closely related to GC249 and GC250 in Changsha, China, and YL201 is in the same subclade as the strains from Chengdu and Beijing, China. These results illustrated that the emergence of dual-resistant FC428-related strains in Guangdong Province is the result of multiregional population exchanges. Meanwhile, FC428 has increased fitness (19); thus, the spread of dual-resistant FC428-related clones might have a huge impact on ceftriaxone and azithromycin efficacy. Overall, we should track the prevalence of dual-resistant clones in future surveillance to effectively curb the large-scale spread of dangerous strains.

Because surveillance of antibiotic resistance requires a series of complex processes, including strain isolation, identification, culture, and AST, which take a long time, genotyping and genome analysis are well established for the investigation of gonococcal transmission and AMR prediction (20). Among them, MLST and ngSTAR are two simple methods to indicate phylogenetic relationships and antibiotic susceptibility. As reported, the MLST ST1901, ST1903, and ST7363 genotypes identified in our study suggested consistency with the evolutionary origins of the F89-, H041-, and FC428-related clones (21), which was further confirmed by whole-genome sequencing (WGS) analysis. For ngSTAR analysis of resistant genes, mutant copies of *ponA*, the *mtrR* efflux pump, *porB*, *gyrA*, and *parC* are correlated with cephalosporin, azithromycin, ciprofloxacin, penicillin, and tetracycline resistance, respectively (15–17). The mosaic *penA*-60.001 allele encodes a mosaic PBP2 causing resistance to extended-spectrum cephalosporins (ESCs) (22), and 23S rRNA-1 mutation decreases macrolide binding to the 50S ribosome and ultimately induces azithromycin resistance, especially at moderate to high antibiotic resistance levels (14). As mosaic *penA* and 23S rRNA mutations are two main contributors to high-level dual resistance strains, genotyping of these two genes might be implemented for gonorrhea epidemic prevention and control.

In conclusion, FC428-related clones coexhibiting azithromycin resistance and high-level dual-resistant clones (such as A2735, F89, and H041) have circulated in Guangdong, China. The capacity for AST and AMR molecular analysis should be strengthened in daily epidemiological surveillance.

## MATERIALS AND METHODS

### Strain collection, cultivation, and preservation.

The Dermatology Hospital of Southern Medical University participates in the World Health Organization (WHO) Gonococcal Antimicrobial Surveillance Program (GASP). All isolates were guaranteed to be N. gonorrhoeae through Gram staining and oxidase, catalase, and sugar fermentation tests recommended by the WHO. N. gonorrhoeae strains were cultured on Thayer-Martin (TM) plates for 18 h in a 37°C incubator with 5% CO_2_. Strains were stored in fetal bovine serum containing 10% dimethyl sulfoxide (DMSO) in liquid nitrogen.

### N. gonorrhoeae isolates and antimicrobial susceptibility testing.

We collected 634, 758, 1,633, and 1,088 strains in 2016, 2017, 2018, and 2019, respectively, from Guangdong Province, China. A total of 4,113 strains were collected for AST, and five high-level dual-resistant isolates (SS36 and SS43 in 2016, DG17067 in 2017, GD69 in 2018, and SS76 in 2019) were identified for further genome-wide testing. AST was conducted using the agar dilution method, according to the WHO recommendations, and details of the experiments are provided below. Briefly, N. gonorrhoeae strains were cultured overnight (for approximately 18 h), adjusted to a 0.5 McFarland standard suspension, and then inoculated onto the surface of antimicrobial agar plates. WHO reference strains D, G, J, L, K, and P were used as quality controls.

### Whole-genome sequencing, genotyping, and AMR analysis.

DNA from the five strains was extracted, sequenced, and assembled by the Beijing Genomics Institute (BGI). The BGISEQ-500 platform was used for WGS. The complete genomic sequences of the five dual-resistant isolates were uploaded to NCBI. The other raw reads were downloaded from the Sequence Read Archive (SRA) database, excluding a subset of strains for which complete genomes were available. These additional public genomes used for comparison were the dual-resistant and FC428-related strains that have been reported globally thus far.

The sequenced reads were preprocessed using fastp v0.20.1 (23) to remove adaptors and low-quality reads. The assembled contigs were generated using SPAdes v3.13 (24) and then used for N. gonorrhoeae sequence typing for antimicrobial resistance (ngSTAR), N. gonorrhoeae multiantigen sequence typing (ngMAST), and multilocus sequence typing (MLST) (25). Finally, the ABRicate tool was used to predict AMR virulence profiles (26).

### Phylogenetic analysis.

In detail, FC428 was set as the reference, and Snippy v4.6 (27) was used for full-length alignment, which was then used as an input into Gubbins v2.3.1 with default parameters to predict and filter regions of homologous recombination (28). The default parameters of Gubbins were as follows: minimum single-nucleotide polymorphisms (SNPs) to identify a recombination block (default, 3); minimum window size (default, 100); and maximum window size (default, 10,000). Then, the filtered alignment to the core polymorphic sites was reduced using SNP-sites v2.5.1 (29), and a maximum likelihood tree was generated using RAxML-NG v1.0.3 with 1,000 bootstrap replicates and a best-fit model (TPMu1f) using the core alignment (containing 1,558 core polymorphic sites) (30, 31). Finally, the tree was visualized and midrooted using iTOL v6.1.1 (32) (Fig. 1).

### Data availability.

The complete genomic sequences of the five dual-resistant isolates are available at NCBI under BioProject accession number PRJNA874857.
